# Butyrate alleviates high fat diet-induced obesity through activation of adiponectin-mediated pathway and stimulation of mitochondrial function in the skeletal muscle of mice

**DOI:** 10.18632/oncotarget.11267

**Published:** 2016-08-12

**Authors:** Jian Hong, Yimin Jia, Shifeng Pan, Longfei Jia, Huifang Li, Zhenqiang Han, Demin Cai, Ruqian Zhao

**Affiliations:** ^1^ Key Laboratory of Animal Physiology & Biochemistry, Nanjing Agricultural University, Nanjing, P. R. China; ^2^ Jiangsu Collaborative Innovation Center of Meat Production and Processing, Quality and Safety Control, Nanjing, P. R. China; ^3^ College of Life Science and Technology, Yancheng Teachers University, Yancheng, P. R. China

**Keywords:** mitochondria function, adipoR1, adipoR2, sodium butyrate, obesity, Pathology Section

## Abstract

Dietary supplementation of butyrate can prevent diet-induced obesity through increasing mitochondrial function in mice, yet the up-stream signaling pathway remains elusive. In this study, weaned mice were divided into two groups, fed control (CON) and high-fat diet (HF, 45% energy from fat), respectively, for 8 weeks. HF-induced obese mice, maintained on HF diet, were then divided into two groups; HFB group was gavaged with 80 mg sodium butyrate (SB) per mice every other day for 10 days, while the HF group received vehicle. It was shown that five gavage doses of SB significantly alleviated HF diet-induced obesity and restored plasma glucose, insulin and leptin to control levels. Muscle contents of ADP and AMP were significantly increased, which was associated with enhanced mitochondrial oxidative phosphorylation and up-regulated expression of fatty acid oxidation enzymes and uncoupling proteins, UCP2 and UCP3 in the skeletal muscle. SB significantly enhanced the expression of adiponectin receptors (adipoR1/2) and AMP kinase (AMPK), while diminished the expression of histone deacetylase 1 (HDAC1). Higher H3K9Ac, a gene activation histone mark, was detected on the promoter of *Adipor1/2*, *Ucp2* and *Ucp3* genes that were activated in the muscle of SB-treated obese mice. Our results indicate that short-term oral administration of SB can alleviate diet-induced obesity and insulin resistance in mice through activation of adiponectin-mediated pathway and stimulation of mitochondrial function in the skeletal muscle.

## INTRODUCTION

Skeletal muscles comprise approximately 50% of the body weight and account for ~80% of insulin-stimulated glucose uptake [1, 2], therefore playing critical roles in whole-body energy balance and glucose homeostasis [3]. High-fat diet-induced obesity often causes insulin resistance [4, 5], which is associated with decreased mitochondrial oxidative capacity and ATP synthesis in the skeletal muscle [6-9]. Conversely, improved skeletal muscle mitochondrial function contributes to enhanced fatty acids β-oxidation and higher insulin sensitivity [10-12]. Therefore, mitochondrial function in skeletal muscle serves as a target for the treatment of high-fat diet-induced obesity and insulin resistance.

Butyrate is a short-chain fatty acid produced from anaerobic bacteria fermentation of dietary fibers in the colon [13]. Butyrate functions not only as a nutrient, but also as a signal to regulate various cellular activities [14]. Firstly, butyrate acts as a histone deacetylase (HDAC) inhibitor to participate in the epigenetic gene regulation [15]; Secondly, butyrate can activate its G protein-coupled receptors, GPR41 and GPR43, to regulate the metabolism and cell fate [16]. Dietary supplementation of butyrate has been shown to prevent high-fat diet-induced obesity and insulin resistance in mice [17-19] or non-alcoholic fatty liver disease in rats [20], through a mechanism related to promotion of energy expenditure and induction of mitochondrial function. In these studies, butyrate was administered together with the high-fat diet for prolonged periods during the induction of disease models to demonstrate its preventive actions. However, the efficacy of butyrate in the treatment of obesity and insulin resistance in established obese model was only lightly mentioned in one study without further mechanistic analysis [19].

Adiponectin is an adipocyte-derived cytokine with a prominent function in improving insulin sensitivity [21, 22], via targeting on mitochondrial functions [23, 24]. The anti-obesity and insulin-sensitizing actions of adiponectin are mediated by its receptors occurring in two isoforms, adipoR1 and adipoR2 [25]. The down-stream signaling of adiponectin receptors involves activation of 5′-adenosine monophosphate-activated protein kinase (AMPK) [26] and peroxisome proliferator-activated receptor-α (PPARα) [27], which subsequently induces mitochondria biogenesis and enhances fatty acid oxidation [28]. Nevertheless, it has not been explored whether adiponectin-mediated pathway is involved in the anti-obesity action of butyrate.

Thus, here we test our hypothesis that adiponectin-mediated pathway contributes to the therapeutic effects of butyrate on a high-fat diet-induced obese mouse model. We show that a short-term oral administration of sodium butyrate was able to alleviate obesity and increase insulin sensitivity through promotion of skeletal muscle mitochondrial function and fatty acid oxidation. Furthermore, we provide evidences that HDAC1 inhibition and histone hyperacetylation on the promoter of genes encoding adiponectin receptors and uncoupling proteins may contribute to butyrate activity.

## RESULTS

### Sodium butyrate reduces body weight gain and improves glucose tolerance

**Figure 1:**
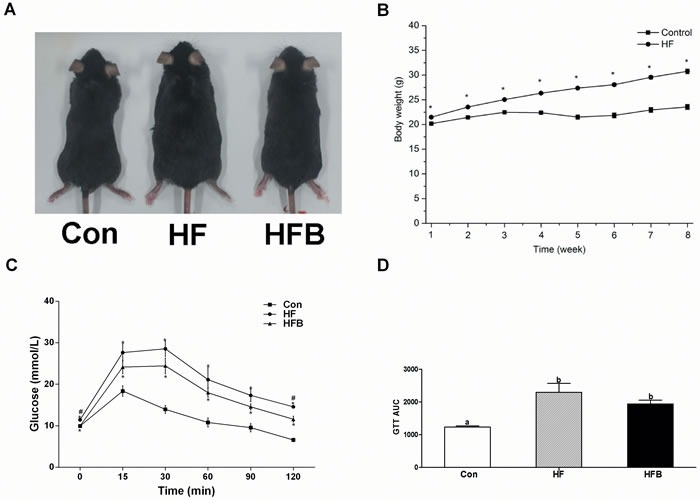
Sodium butyrate reduces body weight gain and improves glucose tolerance **A.** Phenotype of C57BL/6 mice. **B.** Changes in body weight in C57BL/6 mice during 8 weeks’ high-fat diet feeding. **C.** Mean blood glucose levels following GTT (*n* = 8). **D.** Area under the glucose-time curve (AUC). Control: normal control diet, HF: high fat diet, HFB: high fat diet gavage with Sodium Butyrate. The results were expressed as means ± SEM, * indicates the differences compared to the control and # indicates the differences between HF and HFB. Mean values not sharing the same letters are significantly different, *P* < 0.05.

The obese mouse model was established 8 weeks after feeding the high-fat diet, with significantly (*P* < 0.05) higher body weight compared to the mice fed control diet (Figure 1A, 1B). Five gavage doses of SB alleviated glucose intolerance (Figure 1C, 1D). The body weight and the epididymal fat mass were significantly (*P* < 0.05) reduced in HFB group compared to HF group (Table 2).

**Table 1:** The primer of genes for RT-PCR, mtDNA copy number, and ChIP analysis

Primer names	Sequences	Used for
COX1	ATGTTCTATCAATGGGAGC	mRNA quantification
	TCTGAGTAGCGTCGTGGT	
COX2	AGACGAAATCAACAACCC	mRNA quantification
	GGAAGTTCTATTGGCAGA	mtDNA copy number
COX3	CGAAACCACATAAATCAAG	mRNA quantification
	AGTAGGCAAACAATAAGGA	
ND1	CACTATTCGGAGCTTTACG	mRNA quantification
	TGTTTCTGCTAGGGTTGA	
ND2	ACAACCCATCCCTCACTC	mRNA quantification
	ATTTTGGTAAGAATCCTGTT	
ND3	CCTAACGCTAATTCTAGTTG	mRNA quantification
	GACGTGCAGAGCTTGTAG	
ND4	TCCTCAGTTAGCCACATAGCA	mRNA quantification
	AGGCAGAATAGGAGTGATGATG	
ND4L	CTATCACTTCTAGGGACACTT	mRNA quantification
	TAGGGCTAGTCCTACAGC	
ND5	ATAACCGCATCGGAGACA	mRNA quantification
	TGGTAGTCATGGGTGGAG	
ND6	ACAAAGATCACCCAGCTA	mRNA quantification
	GGAGTTATGTTGGAAGGA	
ATP6	AACCTGGTGAACTACGAC	mRNA quantification
	GATGTTACTGTTGCTTGAT	
ATP8	ATGCCACAACTAGATACAT	mRNA quantification
	TAGTGATTTTGGTGAAGG	
CYTB	CTGTTCGCAGTCATAGCC	mRNA quantification
	AAGAATCGGGTCAAGGTG	
PGC-1α	ACACCGCAATTCTCCCTTGT	mRNA quantification
	CGGCGCTCTTCAATTGCTTT	
UCP2	ATCTGGTCATTGTGTTAGGT	mRNA quantification
	GGTCTCTGCTATGCTGTT	
UCP3	ACACTTCCTCCTGCTCTC	mRNA quantification
	AGTCCATTCTGTCCTTCCA	
CPT-1b	CAACACTACACGCATCCC	mRNA quantification
	CACTCTACCCTTCCTCCTG	
Leptin receptor	TGTAAACTGGGACATAGAG	mRNA quantification
	AGGGGTTCTTAGGTAATG	
AdipoR1	GCTGAAAGACAACGACTACC	mRNA quantification
	GTCAAGATTCCCAGAAAGAG	
AdipoR2	GTGTTTTCTTGGTCGTCG	mRNA quantification
	TGCGTCTGGCTGCTGATA	
PPARa	GGGTGGTTGAATCGTGAG	mRNA quantification
	CTTCTCCTTGCCTTTTGC	
PPIA	GCAAGACCAGCAAGAAGA	mRNA quantification
	CAGTGAGAGCAGAGATTACA	mtDNA copy number
AdipoR1	TTCCATACATAGCATACCAG	ChIP
	CATCCTATCTCAGCCTTT	
AdipoR2	GAATAGCAGCACTTTTGG	ChIP
	CGGGCTTTCAGTATGTCT	
UCP2	CGGGGCTAAGGAGGATAA	ChIP
	CCTCAGCGAGATAATGGC	
UCP3	CAAGCCTAAGGGGTAAAG	ChIP
	ACAAATTGGGCATGTCGT	

**Table 2:** Body, liver, gastrocnemius muscle and epididymal fat weight

Parameters	Control (*n* = 8)	HF (*n* = 8)	HFB (*n* = 8)
Initial body weight^1^ (g)	23.65 ± 0.47c	31.72 ± 0.62a	31.35 ± 0.80a
Final body weight^2^ (g)	23.20 ± 0.53c	32.46 ± 0.50a	28.35 ± 0.58b
Liver weight (g)	1.10 ± 0.03b	1.24 ± 0.03a	1.08 ± 0.04b
Gastrocnemius muscle weight (g)	0.29 ± 0.01b	0.35 ± 0.01a	0.32 ± 0.02ab
Epididymal fat weight (g)	0.43 ± 0.04c	1.33 ± 0.13a	0.81 ± 0.08b
Liver index	4.76 ± 0.13a	3.84 ± 0.10b	3.83 ± 0.13b
Gastrocnemius muscle index	1.26 ± 0.08a	1.07 ± 0.04b	1.13 ± 0.06ab
Epididymal fat index	1.83 ± 0.15c	4.06 ± 0.37a	2.83 ± 0.22b

1 Body weight before SB treatment

2 Body weight after SB treatment.

Data are means ± SEM.

Mean values not sharing the same letters are significantly different, *P* < 0.05.

HF, high-fat diet; HFB, high-fat diet gavaged with sodium butyrate.

### Sodium butyrate restores plasma level of insulin and leptin and reduces lipid deposition in the muscle

Plasma concentration of glucose, insulin and leptin was significantly (*P* < 0.05) elevated in HF group, which was completely restored to control levels by SB treatment. Plasma levels of Tch and HDL-c were significantly (*P* < 0.05) higher in HF group, which remained high after SB treatment. Plasma concentrations of TG, LDL-c and NEFA were not affected by high-fat diet or SB treatment (Table 3). Muscle content of TG and Tch was significantly higher (*P* < 0.05) in HF mice, which was significantly (*P* < 0.05) reduced by SB treatment (Table 4).

**Table 3:** The biochemical and hormone parameters in plasma

Parameters	Control (*n* = 8)	HF (*n* = 8)	HFB (*n* = 8)
GLU (mmol/L)	12.36 ± 0.59b	14.69 ± 0.59a	11.43 ± 0.53b
TG (mmol/L)	0.20 ± 0.04	0.27 ± 0.02	0.24 ± 0.02
Tch (mmol/L)	2.51 ± 0.13b	3.16 ± 0.06a	3.22 ± 0.10a
HDLc (mmol/L)	1.72 ± 0.11b	2.42 ± 0.06a	2.41 ± 0.11a
LDLc (mmol/L)	0.52 ± 0.05	0.45 ± 0.02	0.50 ± 0.03
NEFA (μmol/L)	858.4 ± 30.09	824.6 ± 68.19	887.4 ± 44.22
Insulin (ng/mL)	1.51 ± 0.17b	2.39 ± 0.30a	1.25 ± 0.09b
Leptin (ng/mL)	1.45 ± 0.19b	3.71 ± 0.62a	1.50 ± 0.26b

Data are means ± SEM.

Mean values not sharing the same letters are significantly different, *P* < 0.05.

HF, high-fat diet; HFB, high-fat diet gavaged with sodium butyrate; GLU, glucose; TG, triglyceride; Tch, total cholesterol; LDLc, LDL-cholesterol; HDL-C, HDL-cholesterol; NEFA, non-esterified fatty acid.

**Table 4:** The triglyceride, total cholesterol and ATP, AMP, ADP concentrations in gastrocnemius muscle

Parameters	Control (*n* = 8)	HF (*n* = 8)	HFB (*n* = 8)
TG (ng/mg)	7.30 ± 1.28a	16.34 ± 2.88b	10.34 ± 0.98c
Tch (ng/mg)	1.49 ± 0.26a	3.32 ± 0.59b	2.10 ± 0.20c
ATP (ng/mg)	3.14 ± 0.53a	4.45 ± 0.30ab	5.36 ± 0.50b
ADP (ng/mg)	1.03 ± 0.13a	1.52 ± 0.10a	2.02 ± 0.24b
AMP (ng/mg)	0.31 ± 0.01a	0.33 ± 0.01a	0.42 ± 0.05b
Energy charge	0.80 ± 0.01	0.83 ± 0.01	0.82 ± 0.01

Data are means ± SEM.

Mean values not sharing the same letters are significantly different, *P* < 0.05.

HF, high-fat diet; HFB, high-fat diet gavaged with sodium butyrate; TG, triglyceride; Tch, total cholesterol; ATP, adenosine 5′-triphosphate; ADP, adenosine 5′-diphosphate; AMP, adenosine 5′-phosphate; Energy charge = ([ATP] +1/2[ADP])/([ATP] + [ADP] + [AMP]).

### Sodium butyrate improves mitochondrial function and fatty acid β-oxidation

**Figure 2:**
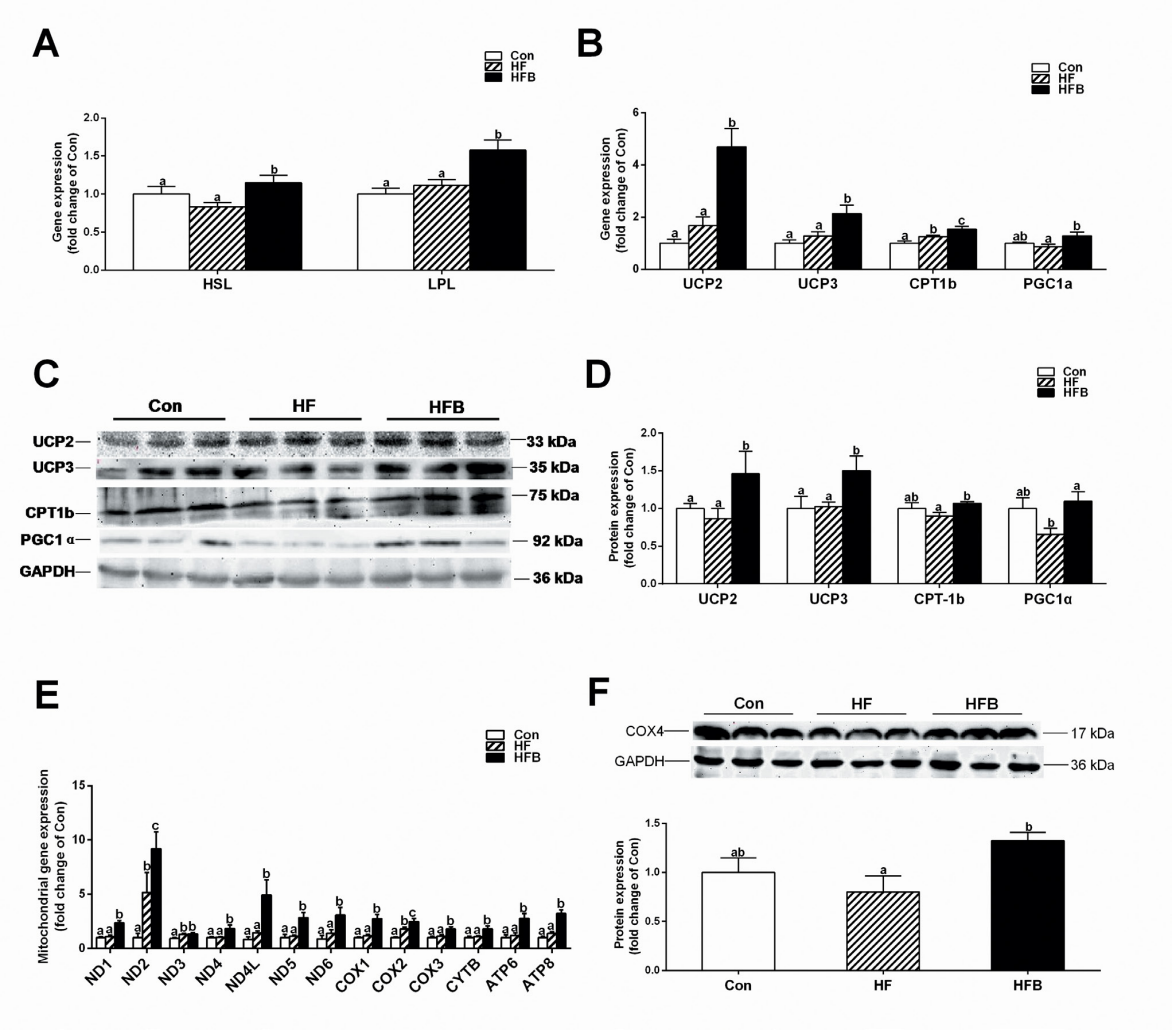
Sodium butyrate improves mitochondrial function and fatty acid β-oxidation **A.** The mRNA expression of *Hsl* and *Lpl* in gastrocnemius muscle (*n* = 10). **B.** The mRNA expression of key genes involved in mitochondrial thermogenesis and fatty acid β-oxidation in gastrocnemius muscle (*n* = 10). **C.**, **D.** The protein expression of key genes involved in mitochondrial thermogenesis and fatty acid β-oxidation in gastrocnemius muscle (*n* = 6). **E.** The mRNA expression of mtDNA-encoded genes involved in oxidative phosphorylation (OXPHOS) in gastrocnemius muscle (*n* = 10). **F.** The protein expression of COX4 in gastrocnemius muscle (*n* = 6). The results were expressed as means ± SEM. Mean values not sharing the same letters are significantly different, *P* < 0.05.

Although myofiber types were not affected (data not shown), muscle content of ADP and AMP was significantly (*P* < 0.05) increased, and that of ATP was numerically increased in SB-treated obese mice, as compared to Con and HF counterparts (Table 4). The reduced muscle lipid content in HFB group was associated with a significant (*P* < 0.05) up-regulation of mRNA expression for hormone sensitive lipase (*Hsl*) and lipoprotein lipase (*Lpl*) (Figure 2A). Moreover, key genes involved in mitochondrial thermogenesis and fatty acid β-oxidation, such as uncoupling protein 2 (UCP2), uncoupling protein 3 (UCP3), carnitine palmitoyl transferase Ib (CPT-1b) and PGC1α, were all increased significantly (*P* < 0.05) at both mRNA (Figure 2B) and protein levels (Figure 2C, 2D). Furthermore, although mtDNA copy number was not affected (data not shown), 12 out of 13 mtDNA-encoded genes (Figure 2E) involved in oxidative phosphorylation (OXPHOS), as well as COX4 protein (Figure 2F) were significantly (*P* < 0.05) up-regulated in the HFB group compared to control and HF groups.

### Sodium butyrate activates adiponectin signaling pathway

**Figure 3:**
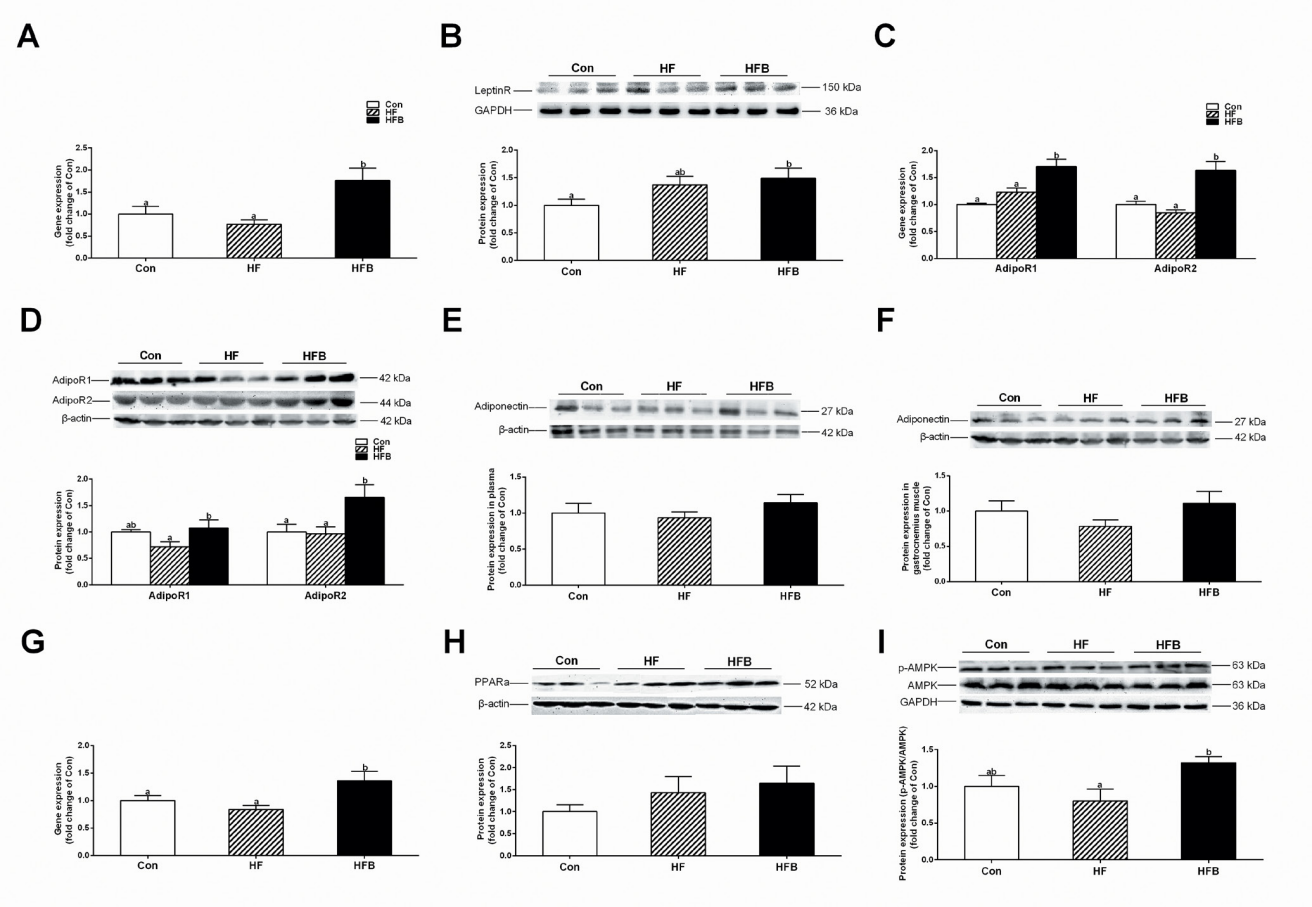
Sodium butyrate activates adiponectin signal pathway in gastrocnemius muscle **A.** The mRNA expression of *Leptin receptor* in gastrocnemius muscle (*n* = 10). **B.** The protein expression of leptin rectpor in gastrocnemius muscle (*n* = 6). **C.** The mRNA expression of *Adipor1* and *Adipor2* in gastrocnemius muscle (*n* = 10). **D.** The protein expression of adiponectin rectpors in gastrocnemius muscle (*n* = 6). **E.** The protein expression of adiponectin in plasma (*n* = 6). **F.** The protein expression of adiponectin in gastrocnemius muscle (*n* = 6). **G.** The mRNA expression of *Ppara* in gastrocnemius muscle (*n* = 10). **H.** The protein expression of PPARa in gastrocnemius muscle (*n* = 6). **I.** The protein expression of p-AMPK in gastrocnemius muscle (*n* = 6); Control: normal control diet, HF: high fat diet, HFB: high fat diet gavage with Sodium Butyrate. The results were expressed as means ± SEM, Mean values not sharing the same letters are significantly different, *P* < 0.05.

SB treatment significantly (*P* < 0.05) increased the mRNA expression of *Leptin receptor* (Figure 3A), yet the protein content was not altered (Figure 3B). In contrast, adipoR1 and adipoR2 were significantly (*P* < 0.05) increased after SB treatment at both mRNA (Figure 3C) and protein levels (Figure 3D), although adiponectin content in the plasma (Figure 3E) and gastrocnemius muscle (Figure 3F) did not change. Accordantly, the two main down-stream signaling pathways of adiponectin receptors, PPARα and AMPK, were activated. PPARα was up-regulated at the level of mRNA (Figure 3G), but not the protein (Figure 3H). SB treatment significantly (*P* < 0.05) increased the protein content of phosphorylated AMPK (p-AMPK), leading to enhanced ratio of p-AMPK/AMPK (Figure 3I).

### Sodium butyrate suppresses HDAC1 expression and modifies histone acetylation

**Figure 4:**
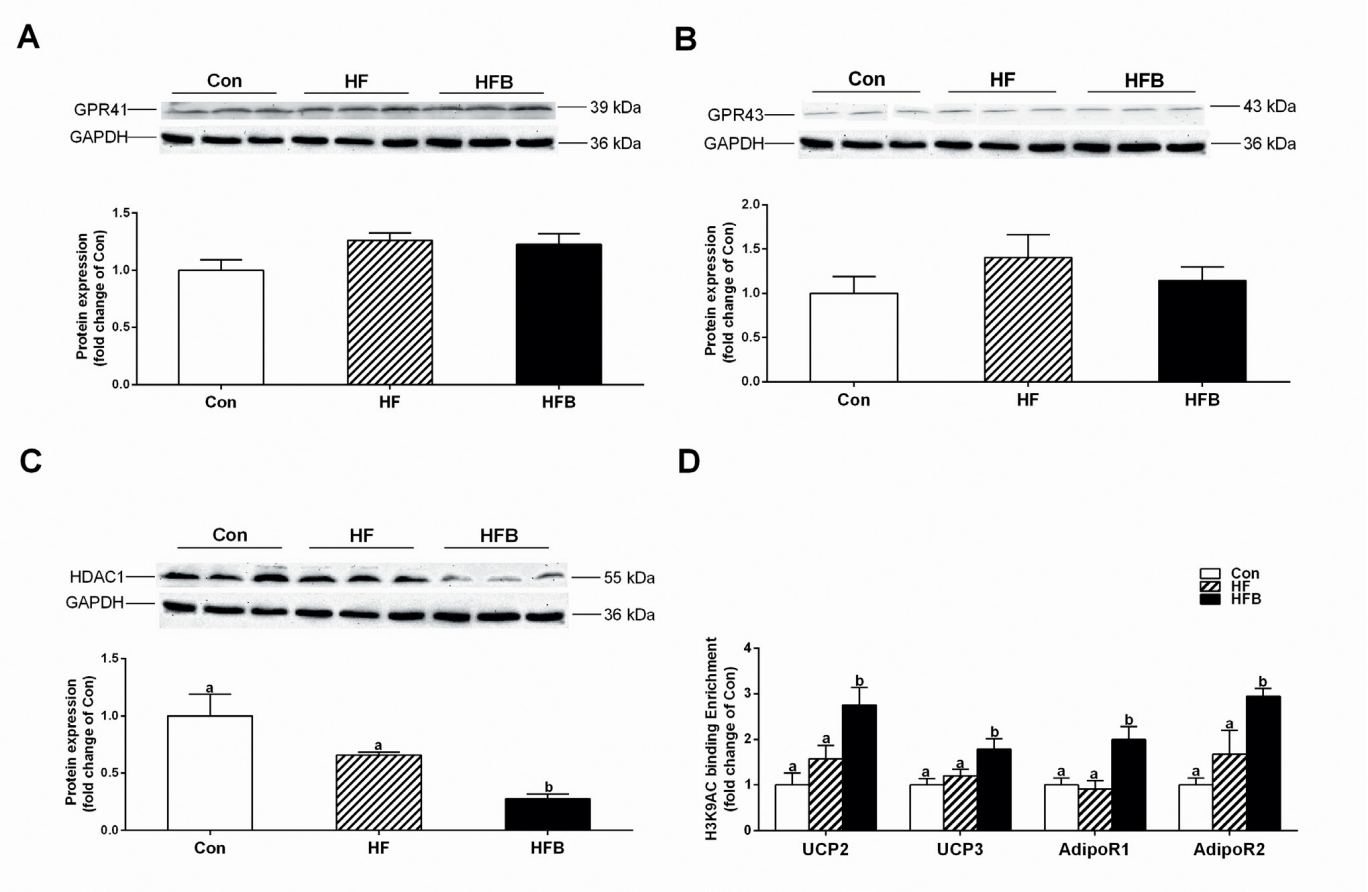
Sodium butyrate suppresses HDAC1 expression and modifies histone acetylation **A.** The protein expression of GPR41 in gastrocnemius muscle (*n* = 6). **B.** The protein expression of GPR43 in gastrocnemius muscle (*n* = 6). **C.** The protein expression of HDAC1 in gastrocnemius muscle (*n* = 6). **D.** Chromatin immunoprecipitation (ChIP) analyses of histone modifications on the gene promoter of *Adipor1, Adipor2, Ucp2 and Ucp3* (*n* = 4). Control: normal control diet, HF: high fat diet, HFB: high fat diet gavage with Sodium Butyrate. The results were expressed as means ± SEM. Mean values not sharing the same letters are significantly different, *P* < 0.05.

Butyrate acts as a histone deacetylase (HDAC) inhibitor or through binding to its G protein-coupled receptors, GPR41 and GPR43. The protein content of GPR43 and GPR41 in gastrocnemius muscle was not affected by either high-fat diet or SB treatment (Figure 4A, 4B). However, the expression of HDAC1 was significantly (*P* < 0.05) decreased in HFB group (Figure. 4C). Furthermore, ChIP analysis detected significant increase of H3K9Ac, a hallmark of gene activation, on the promoter of *Adipor1*, *Adipor2*, *Ucp2* and *Ucp3* genes (Figure 4D).

## DISCUSSION

Butyrate has been reported to protect animals from high-fat diet-induced obesity and insulin resistance [17-19, 29-31]. Here, we provide the evidence that short-term oral administration (5 gavage doses in 10 days) of SB is effective in the treatment of HF diet-induced obesity and glucose intolerance, in a different experimental setting. In previous studies, butyrate was administered during the induction of disease models to demonstrate its preventive functions. The efficacy of butyrate in the treatment of established obesity and insulin resistance was lightly mentioned only in one of these studies in which a 5-week dietary butyrate supplementation was shown effective [19]. Several factors may contribute to the disparities in the effective dose and duration of SB administration. Firstly, compared to long-term dietary supplementation of butyrate (5 g·kg^-1^·day^-1^) in other studies [17-19, 29], 5 gavage doses of SB at 80 mg per mice in this study may ensure higher bioavailability. Secondly, in this study 3-week-old male C57BL/6J mice were fed HF diet for 8 weeks to induce obesity, whereas in other studies, older mice (4-8 weeks of age) were used and obesity was induced by feeding high-fat diet for longer period (9-12 weeks) [17-19, 29]. Therefore, compared to the severe obese model in other studies, the model established in this study may resemble a mild obesity or prediabetes condition, which is commonly seen in human society and clinical situations.

The mechanism of butyrate action for obesity and insulin resistance is related to promotion of energy expenditure and induction of mitochondrial function. AMPK and PGC-1α have been suggested to mediate the effects of butyrate to increase fatty acid β-oxidation and enhance mitochondrial uncoupling [17, 19]. In agreement with these previous publications, we also detected significant activation of AMPK and PGC-1α, in association with up-regulated expression of genes involved in lipolysis, fatty acid β-oxidation, mitochondria OXPHOS and mitochondria uncoupling after a short-term oral SB treatment in a HF diet-induced mouse model.

In this study, obesity and glucose intolerance were evident after 8-weeks feeding with high-fat diet, which is indicated by significantly increased body weight, elevated fasting blood glucose level, higher plasma leptin and insulin concentrations, as well as diminished oral glucose tolerance. Mice fed high-fat diet were reported to show significantly decreased expression of mitochondrial biogenesis genes [8] and impaired mitochondrial function and β-oxidation activities in skeletal muscle [7, 9, 32]. Also, high-fat diet fed mice exhibit inhibition of glucose uptake through inactivated AMPK signaling pathways in skeletal muscle [33]. Surprisingly, however, the expression of genes involved in lipolysis, β-oxidation, mitochondrial OXPHOS or mitochondria uncoupling was not altered in obese mice compared to their lean counterparts in this study. It is speculated that, compared to other tissues such as adipose tissue and liver, skeletal muscles are less vulnerable to the influence of high-fat diet in a condition of mild obesity. Nevertheless, it may not be true because muscle lipid contents including TG and Tch were significantly higher in obese mice compared to the control. Therefore, genes responsible for lipid synthesis and transport may be modulated in obese mice. However, this is beyond the scope of this study that was aimed to explore the up-stream signaling pathway of butyrate function.

The most striking effect of SB in this study is to restore the plasma leptin and insulin to control levels. Obviously this attributes to the potential actions of butyrate on adipose tissue and pancreatic β cells that secrete leptin and insulin, respectively. Certainly possible modulation of the clearance or turnover cannot be excluded. However, this study was focused on skeletal muscle, an important target of these hormones. To our disappointment, no consistent responses to SB treatment were detected for insulin receptor (data not shown) or leptin receptor that changed only at the level of mRNA. Unexpectedly, however, both adiponectin receptors, adipoR1 and adipoR2, were found to be up-regulated after SB treatment, at both mRNA and protein levels. Although both leptin and adiponectin are secreted from adipose tissue, the two adipokines behave differently in high-fat diet-induced obesity. Obese mice had significantly elevated plasma leptin level that was completely restored by SB treatment, whereas adiponectin concentration in the plasma and skeletal muscle was not altered in obese mice or affected by SB treatment. This observation implicates different regulatory mechanisms for the secretion and action of leptin and adiponectin, in response to high-fat diet and SB treatment.

The down-stream signaling of adiponectin receptors has been well documented. In this study, activation of AMPK appears to be the main pathway responsible for the beneficial effects of SB on obesity and insulin resistance. However, how SB induces activation of adiponectin receptors has not been elucidated. Several studies have addressed the transcriptional regulation of Adipor1 and Adipor2 genes. Nuclear inhibitory protein (NIP) [34] and activating transcription factor 3 (ATF3) [35] are reported to repress the promoter activity of Adipor1 gene, whereas PPARG agonist, growth hormone and SIRT1-Foxo1 signaling are shown to stimulate the promoter activity of Adipor2 gene [36, 37].

To understand how sodium butyrate up-regulates the expression of adiponectin receptors, we explored both mechanisms known to mediate butyrate action, inhibition of HDAC [15] and activation of GPR41 and GPR43 [16]. It appears that SB exerts its function in this study mainly as an HDAC inhibitor, as neither GPR41 nor GPR43 was modulated by butyrate treatment. It has been shown that butyrate prevents inhibition of promoter activity by suppression of histone deacetylase [19]. In this study, H3K9Ac, a gene activation mark, was found to be highly enriched in the promoter of Adipor1, Adipor2, Ucp2 and Ucp3 genes that are up-regulated by SB treatment. Certainly, the coincidence and the association analysis of all the events observed in the present study are not adequate to draw a comprehensive picture of the signaling pathway underlying butyrate action. A more detailed mechanistic study using loss of function and gain of function strategies is required to delineate the cause and consequences. Also, skeletal muscle is not a sole target for sodium butyrate when administered systematically. The actions of sodium butyrate and the underlying mechanisms in other target tissues could be very different and it merits future investigations how different tissues respond in concert to butyrate to achieve the general beneficial effects on obesity and glucose intolerance.

In summary, this study provides a better understanding of the signaling pathways through which butyrate alleviates obesity and improves glucose tolerance in a high-fat diet-induced obese mouse model. The fact that a short-term oral administration of SB is effective in alleviate diet-induced mild obesity in mice may encourage future investigation of butyrate and its derivatives in the treatment and prevention of obesity-related metabolic disorders in humans.

## MATERIALS AND METHODS

### Animal models and experimental protocols

Thirty six male specific pathogen-free (SPF) C57BL/6J (3-week-old, 8-10 g) mice were obtained from the Comparative Medicine Center of Yangzhou University (Yangzhou, China, certificate of quality is SCXK (Su) 2012-0004) and fed in the Laboratory Animal Center of Jiangsu Province Integrative Medicine Hospital. The mice were housed in a controlled environment (22 ± 3°C, 50-60% relative humidity) with a 12L:12D lighting cycle and allowed to adapt to their environment for one week. Then mice were randomly assigned to two groups; one was fed a control diet (Con, *n* = 12) and another was fed a high-fat diet (HF, 45% energy from fat, *n* = 24) for 8 weeks to establish obesity. The mice were fed ad libitum with free access to water and weighed once per week. HF-induced obese mice maintained on HF diet were divided into two groups (*n* = 12); HFB group was gavaged with 80 mg sodium butyrate (SB) in 1 ml deionized water per mice every other day for 10 days, while the HF group received vehicle. Mice in the control group were kept continually on a normal diet for the whole experiment. The mice were fasted overnight before sampling. The blood and gastrocnemius muscle samples were collected and stored at −80°C. All the procedures were approved by the Animal Ethics Committee of Nanjing Agricultural University, with the project number 2012CB124703. The slaughter and sampling procedures complied with the “Guidelines on Ethical Treatment of Experimental Animals’’ (2006) No. 398 set by the Ministry of Science and Technology, China.

### Glucose tolerance test

Eight mice from each group were used for the glucose tolerance test. They were fasted for 10 h (from 8:30 am till 18:30 pm), and were injected intraperitoneally with 2.5 g/kg glucose (G7021; Sigma). The blood glucose levels before glucose injection (0 min) and 15, 30, 60, 90, and 120 min after glucose injection were determined.

### Plasma concentration of biochemical parameters and hormones

Plasma concentration of glucose (GLU), triglycerides (TG), total cholesterol (Tch), high-density lipoprotein cholesterol (HDL-c), low-density lipoprotein cholesterol (LDL-c) and non-esterified fatty acid (NEFA) was detected with automatic biochemical analyzer (Hitachi 7020; HITACHI) using commercial assay kits (995-18311, 995-33093, 999-33493, 998-09011, 993-39993 and 995-09901, respectively; Wako Pure Chemical Industries, Ltd. Wako). Plasma concentration of insulin (no.96-416) and leptin (no.96-421) were measured by China Biomarker Service, Luminex 200 (no. CNBMSLX200) using Magnetic Bead MAPmate (Merck & Millipore, Darmstadt, Germany) according to the instruction provided by the manufacturer.

### Content of triglycerides and total cholesterol in gastrocnemius muscle

Muscle content of TG (E1013; Applygen Technologies, Inc.) and Tch (E1015; Applygen Technologies, Inc.) was measured using respective commercial assay kits following the manufacturer's instruction.

### ATP, ADP and AMP levels in gastrocnemius muscle

Muscle content of ATP, ADP and AMP was determined by high performance liquid chromatography (HPLC) according to previous publications with some modifications [38, 39]. Tissue extracts were prepared from frozen muscle using 0.6 mol/l perchloric acid, and the extracts were centrifuged at 10,000×g for 10 min at 4°C. The ATP (FLAAS), ADP (A5285) and AMP (01930) standards were purchased from Sigma. HPLC was performed with A reverse-phase column (99603, C18, 5 mm, 25064.6 mm, Dikma Technologies Inc.). The column temperature was set at 25°C. For metabolites measurements, a mobile phase consisting of 215 mmol/l KH_2_PO_4_, 1.2 mmol/l tetrabutylammonium bisulfate, 1% acetonitrile (pH 6.0) was used and the flow rate was maintained at 1.0 ml·min^-1^ by a HPLC pump (600E, Waters). Eluted samples were detected at 254 nm with a dual λ absorbance detector (2478, Waters). Calibration curves were prepared by a six-point standard (0.2, 0.1, 0.05, 0.0125 and 0.00625 mg·ml^-1^) of ATP, ADP and AMP in 0.6 mol/l perchloric acid, respectively.

### Determination of mtDNA copy number

Total genomic DNA was isolated from muscle samples and the mtDNA copy number was determined using real-time PCR. For DNA extraction, muscle samples were incubated in a lysis buffer containing 0.5 mmol/l of EDTA, pH 8.0 and 2 mg/ml of proteinase K (Amresco, USA) at 37°C overnight. Primers specific for the control region of mitochondrial DNA (mtDNA) were used for the quantification of mtDNA, whereas primers specific for the nuclear gene peptidylprolyl isomerase A (*Ppia*) were used for standardization. Relative mtDNA copy number was calculated with 2^-ΔΔCt^ method [40].

### RNA isolation and quantitative real-time PCR

Total RNA was extracted from frozen gastrocnemius muscle samples (40 mg) using TRIzol reagent (15596026, Invitrogen) according to the manufacturer's protocol. Two micrograms of RNA were used to generate cDNA by PrimeScript^®^ 1st Strand cDNA Synthesis Kit (D6110A, Takara). *Ppia* was used as an internal control. The resulting cDNA was diluted 1:25 and 2 μL of diluted cDNA was used as template in PCR reactions on a real-time PCR system (Mx3000P, Stratagene, USA). All primers were synthesized by Generay Biotech and listed in Table 1. The 2^-ΔΔCt^ method was used to analyze real-time PCR data [40].

### Western blotting

Total protein was extracted from 40 mg frozen gastrocnemius muscle samples as previously described [41]. Protein concentration was measured with a Pierce BCA Protein Assay kit (23225; Thermo Scientific). Antibodies and their sources are: Adiponectin (BS6961, Bioworld), AdipoR1 (BS6797, Bioworld), AdipoR2 (14361-1-AP, Proteintech), AMPKα112 (BS1009, Bioworld), p-AMPKα1/2 (sc-33524, Santa Cruz), PGC-1α (sc-13067, Santa Cruz), UCP3 (BS2849, Bioworld), UCP2 (BS2917, Bioworld), GPR43 (sc-32906, Santa Cruz), GPR41 (sc-98332, Santa Cruz), CPT-1b (sc-20670, Santa Cruz), COX4 (MB0102, Bioworld), HDAC1 (BS6485, Bioworld), GAPDH (AP0063, Bioworld), β-actin (AP0063, Bioworld). The phosphorylated AMPK was normalized by the total AMPK.

### Chromatin immunoprecipitation assay

Approximately 200 mg of frozen muscle samples were homogenized in liquid nitrogen and resuspended in PBS containing protease inhibitor cocktail (no. 11697498001; Roche). Cross-linking of protein and DNA was performed by adding formaldehyde to a final concentration of 1%, and then the reaction was stopped with 2.5mol/l glycine at room temperature. The reaction mix was centrifuged and the pellets were rinsed with PBS and homogenized in a SDS lysis buffer containing protease inhibitors. Crude chromatin preparations were sonicated on ice to yield DNA fragments of 200 to 500 bp in length and pre-cleared with salmon sperm DNA-treated protein G agarose beads (40 μL, 50% slurry, sc-2003; Santa Cruz). The mixture of pre-cleared chromatin preparations and 2 μg of primary antibody (H3 lysine 9 acetylation, ab4441, Abcam) were incubated overnight at 4°C. A negative control was included with normal IgG. Protein G agarose beads (40 μl, 50% slurry, sc-2003; Santa Cruz) were added to capture the immunoprecipitated chromatin complexes. Finally, DNA fragments were released from the immunoprecipitated complexes by reverse cross-linking at 65°C for 1 h, and quantitative real-time PCR was used to quantify the fragments of target gene promoters with specific primers (see supplementary Table 1) using purified immunoprecipitated DNA as the template.

### Statistical analysis

All data are presented as mean ± SEM. One-way ANOVA with a Bonferroni post hoc test was used to evaluate the statistical significance of differences among 3 groups. A value *P* < 0.05 was considered statistically significant.

## References

[1] de Lange P, Moreno M, Silvestri E, Lombardi A, Goglia F, Lanni A (2007). Fuel economy in food-deprived skeletal muscle: signaling pathways and regulatory mechanisms. FASEB journal.

[2] Shulman GI, Rothman DL, Jue T, Stein P, DeFronzo RA, Shulman RG (1990). Quantitation of muscle glycogen synthesis in normal subjects and subjects with non-insulin-dependent diabetes by 13C nuclear magnetic resonance spectroscopy. The New England journal of medicine.

[3] Lee-Young RS, Bonner JS, Mayes WH, Iwueke I, Barrick BA, Hasenour CM, Kang L, Wasserman DH (2013). AMP-activated protein kinase (AMPK)alpha2 plays a role in determining the cellular fate of glucose in insulin-resistant mouse skeletal muscle. Diabetologia.

[4] Kelley DE, Goodpaster B, Wing RR, Simoneau JA (1999). Skeletal muscle fatty acid metabolism in association with insulin resistance, obesity, and weight loss. The American journal of physiology.

[5] van der Heijden RA, Sheedfar F, Morrison MC, Hommelberg PP, Kor D, Kloosterhuis NJ, Gruben N, Youssef SA, de Bruin A, Hofker MH, Kleemann R, Koonen DP, Heeringa P (2015). High-fat diet induced obesity primes inflammation in adipose tissue prior to liver in C57BL/6j mice. Aging (Albany NY).

[6] Wiesenborn DS, Menon V, Zhi X, Do A, Gesing A, Wang Z, Bartke A, Altomare DA, Masternak MM (2014). The effect of calorie restriction on insulin signaling in skeletal muscle and adipose tissue of Ames dwarf mice. Aging (Albany NY).

[7] Hirabara SM, Curi R, Maechler P (2010). Saturated fatty acid-induced insulin resistance is associated with mitochondrial dysfunction in skeletal muscle cells. Journal of cellular physiology.

[8] Patti ME, Butte AJ, Crunkhorn S, Cusi K, Berria R, Kashyap S, Miyazaki Y, Kohane I, Costello M, Saccone R, Landaker EJ, Goldfine AB, Mun E, DeFronzo R, Finlayson J, Kahn CR (2003). Coordinated reduction of genes of oxidative metabolism in humans with insulin resistance and diabetes: Potential role of PGC1 and NRF1. Proceedings of the National Academy of Sciences of the United States of America.

[9] Kelley DE, He J, Menshikova EV, Ritov VB (2002). Dysfunction of mitochondria in human skeletal muscle in type 2 diabetes. Diabetes.

[10] Galgani JE, Johannsen NM, Bajpeyi S, Costford SR, Zhang Z, Gupta AK, Ravussin E (2012). Role of skeletal muscle mitochondrial density on exercise-stimulated lipid oxidation. Obesity.

[11] Meex RC, Schrauwen-Hinderling VB, Moonen-Kornips E, Schaart G, Mensink M, Phielix E, van de Weijer T, Sels JP, Schrauwen P, Hesselink MK (2010). Restoration of muscle mitochondrial function and metabolic flexibility in type 2 diabetes by exercise training is paralleled by increased myocellular fat storage and improved insulin sensitivity. Diabetes.

[12] Solomon TP, Sistrun SN, Krishnan RK, Del Aguila LF, Marchetti CM, O’Carroll SM, O’Leary VB, Kirwan JP (2008). Exercise and diet enhance fat oxidation and reduce insulin resistance in older obese adults. Journal of applied physiology.

[13] Pryde SE, Duncan SH, Hold GL, Stewart CS, Flint HJ (2002). The microbiology of butyrate formation in the human colon. FEMS microbiology letters.

[14] Berni Canani R, Di Costanzo M, Leone L (2012). The epigenetic effects of butyrate: potential therapeutic implications for clinical practice. Clinical epigenetics.

[15] Davie JR (2003). Inhibition of histone deacetylase activity by butyrate. The Journal of nutrition.

[16] Brown AJ, Goldsworthy SM, Barnes AA, Eilert MM, Tcheang L, Daniels D, Muir AI, Wigglesworth MJ, Kinghorn I, Fraser NJ, Pike NB, Strum JC, Steplewski KM, Murdock PR, Holder JC, Marshall FH (2003). The Orphan G protein-coupled receptors GPR41 and GPR43 are activated by propionate and other short chain carboxylic acids. The Journal of biological chemistry.

[17] Henagan TM, Stefanska B, Fang Z, Navard AM, Ye J, Lenard NR, Devarshi PP (2015). Sodium butyrate epigenetically modulates high-fat diet-induced skeletal muscle mitochondrial adaptation, obesity and insulin resistance through nucleosome positioning. British journal of pharmacology.

[18] Vinolo MA, Rodrigues HG, Festuccia WT, Crisma AR, Alves VS, Martins AR, Amaral CL, Fiamoncini J, Hirabara SM, Sato FT, Fock RA, Malheiros G, dos Santos MF, Curi R (2012). Tributyrin attenuates obesity-associated inflammation and insulin resistance in high-fat-fed mice. American journal of physiology Endocrinology and metabolism.

[19] Gao Z, Yin J, Zhang J, Ward RE, Martin RJ, Lefevre M, Cefalu WT, Ye J (2009). Butyrate improves insulin sensitivity and increases energy expenditure in mice. Diabetes.

[20] Mattace Raso G, Simeoli R, Russo R, Iacono A, Santoro A, Paciello O, Ferrante MC, Canani RB, Calignano A, Meli R (2013). Effects of sodium butyrate and its synthetic amide derivative on liver inflammation and glucose tolerance in an animal model of steatosis induced by high fat diet. PloS one.

[21] Adams AC, Kharitonenkov A (2013). FGF21 drives a shift in adipokine tone to restore metabolic health. Aging (Albany NY).

[22] Civitarese AE, Ukropcova B, Carling S, Hulver M, DeFronzo RA, Mandarino L, Ravussin E, Smith SR (2006). Role of adiponectin in human skeletal muscle bioenergetics. Cell metabolism.

[23] Yamauchi T, Kamon J, Waki H, Terauchi Y, Kubota N, Hara K, Mori Y, Ide T, Murakami K, Tsuboyama-Kasaoka N, Ezaki O, Akanuma Y, Gavrilova O, Vinson C, Reitman ML, Kagechika H (2001). The fat-derived hormone adiponectin reverses insulin resistance associated with both lipoatrophy and obesity. Nature medicine.

[24] Fruebis J, Tsao TS, Javorschi S, Ebbets-Reed D, Erickson MR, Yen FT, Bihain BE, Lodish HF (2001). Proteolytic cleavage product of 30-kDa adipocyte complement-related protein increases fatty acid oxidation in muscle and causes weight loss in mice. Proceedings of the National Academy of Sciences of the United States of America.

[25] Yamauchi T, Nio Y, Maki T, Kobayashi M, Takazawa T, Iwabu M, Okada-Iwabu M, Kawamoto S, Kubota N, Kubota T, Ito Y, Kamon J, Tsuchida A, Kumagai K, Kozono H, Hada Y (2007). Targeted disruption of AdipoR1 and AdipoR2 causes abrogation of adiponectin binding and metabolic actions. Nature medicine.

[26] Yamauchi T, Kamon J, Minokoshi Y, Ito Y, Waki H, Uchida S, Yamashita S, Noda M, Kita S, Ueki K, Eto K, Akanuma Y, Froguel P, Foufelle F, Ferre P, Carling D (2002). Adiponectin stimulates glucose utilization and fatty-acid oxidation by activating AMP-activated protein kinase. Nature medicine.

[27] Yamauchi T, Kamon J, Waki H, Imai Y, Shimozawa N, Hioki K, Uchida S, Ito Y, Takakuwa K, Matsui J, Takata M, Eto K, Terauchi Y, Komeda K, Tsunoda M, Murakami K (2003). Globular adiponectin protected ob/ob mice from diabetes and ApoE-deficient mice from atherosclerosis. The Journal of biological chemistry.

[28] Yamauchi T, Kamon J, Ito Y, Tsuchida A, Yokomizo T, Kita S, Sugiyama T, Miyagishi M, Hara K, Tsunoda M, Murakami K, Ohteki T, Uchida S, Takekawa S, Waki H, Tsuno NH (2003). Cloning of adiponectin receptors that mediate antidiabetic metabolic effects. Nature.

[29] den Besten G, Bleeker A, Gerding A, van Eunen K, Havinga R, van Dijk TH, Oosterveer MH, Jonker JW, Groen AK, Reijngoud DJ, Bakker BM (2015). Short-Chain Fatty Acids Protect Against High-Fat Diet-Induced Obesity via a PPARgamma-Dependent Switch From Lipogenesis to Fat Oxidation. Diabetes.

[30] Li HP, Chen X, Li MQ (2013). Butyrate alleviates metabolic impairments and protects pancreatic beta cell function in pregnant mice with obesity. International journal of clinical and experimental pathology.

[31] Lin HV, Frassetto A, Kowalik EJ, Nawrocki AR, Lu MM, Kosinski JR, Hubert JA, Szeto D, Yao X, Forrest G, Marsh DJ (2012). Butyrate and propionate protect against diet-induced obesity and regulate gut hormones via free fatty acid receptor 3-independent mechanisms. PloS one.

[32] Chen LL, Zhang HH, Zheng J, Hu X, Kong W, Hu D, Wang SX, Zhang P (2011). Resveratrol attenuates high-fat diet-induced insulin resistance by influencing skeletal muscle lipid transport and subsarcolemmal mitochondrial beta-oxidation. Metabolism.

[33] Choi WH, Um MY, Ahn J, Jung CH, Ha TY (2014). Long-term intake of rice improves insulin sensitivity in mice fed a high-fat diet. Nutrition.

[34] Sun X, He J, Mao C, Han R, Wang Z, Liu Y, Chen Y (2008). Negative regulation of adiponectin receptor 1 promoter by insulin via a repressive nuclear inhibitory protein element. FEBS letters.

[35] Park HJ, Kang YM, Kim CH, Jung MH (2010). ATF3 negatively regulates adiponectin receptor 1 expression. Biochemical and biophysical research communications.

[36] Liang X, Hu M, Rogers CQ, Shen Z, You M (2011). Role of SIRT1-FoxO1 signaling in dietary saturated fat-dependent upregulation of liver adiponectin receptor 2 in ethanol-administered mice. Antioxidants & redox signaling.

[37] Sun X, Han R, Wang Z, Chen Y (2006). Regulation of adiponectin receptors in hepatocytes by the peroxisome proliferator-activated receptor-gamma agonist rosiglitazone. Diabetologia.

[38] Goldman N, Chen M, Fujita T, Xu Q, Peng W, Liu W, Jensen TK, Pei Y, Wang F, Han X, Chen JF, Schnermann J, Takano T, Bekar L, Tieu K, Nedergaard M (2010). Adenosine A1 receptors mediate local anti-nociceptive effects of acupuncture. Nature neuroscience.

[39] Smith DJ, Stokes BO, Boyer PD (1976). Probes of initial phosphorylation events in ATP synthesis by chloroplasts. The Journal of biological chemistry.

[40] Livak KJ, Schmittgen TD (2001). Analysis of relative gene expression data using real-time quantitative PCR and the 2(-Delta Delta C(T)) Method. Methods.

[41] Jia Y, Cong R, Li R, Yang X, Sun Q, Parvizi N, Zhao R (2012). Maternal low-protein diet induces gender-dependent changes in epigenetic regulation of the glucose-6-phosphatase gene in newborn piglet liver. The Journal of nutrition.

